# Simplified Anisotropic IVIM Using Spherical Means and an Application in the Placenta

**DOI:** 10.1002/mrm.70598

**Published:** 2026-09-20

**Authors:** Paddy J. Slator, Luke Pleva, Alexandra Crawford, Lucy Higgins, Edward Johnstone, Alexander E. P. Heazell, Daniel C. Alexander, Josephine H. Naish, Kate Duhig

**Affiliations:** ^1^ Cardiff University Brain Research Imaging Centre, School of Psychology Cardiff UK; ^2^ School of Computer Science and Informatics, Cardiff University Cardiff UK; ^3^ BHF Manchester Centre for Heart and Lung Magnetic Resonance Research University of Manchester NHS Foundation Trust Manchester UK; ^4^ Division of Cardiovascular Sciences, Faculty of Biology, Medicine and Health The University of Manchester Manchester UK; ^5^ Maternal and Fetal Health Research Centre Institute of Human Development, University of Manchester Manchester UK; ^6^ St. Mary's Hospital, Manchester University Hospitals NHS Foundation Trust, Manchester Academic Health Science Centre Manchester UK; ^7^ UCL Hawkes Institute, University College London London UK; ^8^ Department of Computer Science University College London London UK; ^9^ Department of Women and Children's Health King's College London London UK

## Abstract

**Purpose:**

To reduce the complexity associated with anisotropic IVIM modeling, we derived two simplified diffusion MRI models that map the apparent diffusivity, relative proportions, and anisotropy of perfusion‐ and diffusion‐related signal components and demonstrated them in placenta scans.

**Methods:**

We introduce two spherical mean anisotropic intravoxel incoherent motion (IVIM) models. The models consist of constrained diffusion tensor compartments, namely “sticks” or “zeppelins,” and we consider the spherical mean of the signal for these compartments. We apply these spherical mean anisotropic IVIM models to placenta MRI scans from 14 control participants to produce perfusion fraction, perfusion‐related pseudo‐diffusivity, diffusion‐related diffusivity, perfusion‐related fractional anisotropy, and diffusion‐related fractional anisotropy maps. We compare our approach to the standard IVIM model and calculate the Bayesian information criterion voxelwise to determine which model best explains the data.

**Results:**

Spherical mean anisotropic IVIM output maps are consistent with standard IVIM, while additionally accounting for and mapping anisotropy of perfusion‐ and diffusion‐related signal components. Spherical mean anisotropic IVIM models provide a better explanation of the data, as indicated by a lower Bayesian information criterion compared to standard IVIM in 10%–20% of voxels, while retaining sensitivity to the same underlying tissue and perfusion characteristics in the remaining voxels.

**Conclusion:**

Our spherical mean anisotropic IVIM approach can disentangle perfusion‐ and diffusion‐related anisotropy without explicitly estimating directionality. Our approach simplifies the calculation of biomarkers reflecting microcirculatory and microstructural changes in anisotropic tissue by employing models of reduced complexity compared to previous methods.

## 1 Introduction

Diffusion MRI (dMRI) quantifies microstructural and microcirculatory properties of tissue by sensitizing the signal to the motion of water diffusion and pseudo‐diffusion. Low *b* values (approximately *b* < 200 s/mm^2^) encode sensitivity to faster pseudo‐diffusion in capillaries and blood vessels, and higher *b* values encode sensitivity to diffusion in tissue. The intravoxel incoherent motion (IVIM) model, which for clarity we refer to as *standard IVIM* throughout, separates and quantifies diffusion‐ and perfusion‐related components of the dMRI signal [1].

However, the standard IVIM model assumes isotropic signal attenuation within imaged tissue, overlooking macroscopic anisotropy in both components. This anisotropy arises in diffusion‐related signal components due to structures such as white matter fiber tracts, and in perfusion‐related signal components due to coherent vasculature architectures, such as those found in muscle [2]. Extensions to standard IVIM that additionally account for macroscopic anisotropy in diffusion‐ and perfusion‐related signal components have been proposed and applied in muscle [2], brain [3, 4, 5, 6], heart [7], kidney [8, 9, 10, 11, 12], placenta [13], and peripheral nerves [14]. These *anisotropic IVIM* models offer the opportunity for new imaging biomarkers. For example, they have been used to noninvasively quantify kidney function through estimation of the glomerular filtration rate in patients with kidney cancer [15].

Unlike standard IVIM, which assumes isotropic diffusion in perfusion‐ and diffusion‐related signal components, these models adopt alternative formulations, often using diffusion tensors, to capture anisotropic diffusion. Karampinos et al. reformulated the standard IVIM model to assume that capillaries are distributed according to a Fisher distribution which can be parameterized between 0 (isotropic capillary orientation) and infinity (fully anisotropic capillary orientation) [2]. Abdullah et al. replaced the scalar diffusivities of the standard IVIM model with tensor representations [7] and used this for simulations; this model can also be fit to data [4]. Hilbert et al. proposed a model in which the diffusivity, perfusion‐related pseudo‐diffusivity, and perfusion fraction are each represented by full tensors. This results in a model with 18 free parameters. To enable robust parameter estimation, the authors made several simplifying assumptions, including constraining some tensors to be symmetric, and employed a multi‐stage fitting procedure. This model was initially demonstrated in the kidney [9] and subsequently applied in the brain [3, 5]. Notohamiprodjo et al. introduced a combined IVIM–DTI approach in which standard IVIM and DTI models are fitted separately across multiple gradient directions, enabling estimation of diffusion‐ and perfusion‐related tensors [8, 10, 11]. All of the aforementioned methods share the limitation of being highly parameterized and challenging to fit.

The placenta has a unique architecture, with separate but adjacent maternal and fetal circulations. Fetal blood flows through a branching vascular tree, from large stem villi down to capillary‐rich terminal villi, while maternal blood moves through tortuous intervillous spaces outside the fetal villi. Both circulations must transport blood across the placenta: deoxygenated fetal blood enters at the chorionic plate, reaches the capillaries, and returns oxygenated; oxygenated maternal blood enters via spiral arteries, flows through intervillous spaces, and drains into decidual veins deoxygenated. These flow patterns give rise to various anisotropic structures—both microanisotropic and macroanisotropic—resulting in anisotropic attenuation of the perfusion‐related signal. This was demonstrated using anisotropic IVIM models with constrained tensors (“zeppelins” and “sticks”), which provided a better explanation of placental dMRI signal in the model‐theoretic sense, that is, achieved a lower Bayesian information criterion [13], indicating that anisotropic signal contributions are present and detectable in the placenta.

Spherical mean techniques compute the average dMRI signal across gradient directions. When combined with microstructural models this enables calculation of quantitative metrics while factoring out orientation dependence [16]. Such models generally have fewer parameters and are hence easier to fit, while still accounting for anisotropy. Notably, spherical mean models are particularly well suited to capturing microanisotropy via directionally averaged signals, whereas nonspherical mean models retain directional information and are more appropriate for resolving coherent macrostructural anisotropy. The *spherical mean technique* (SMT) is a well‐established method in brain imaging based on the spherical average of the signal [16]. Here we introduce two simplified anisotropic IVIM models that adopt a similar spherical mean approach and demonstrate their application in placental MRI.

## 2 Methods

### 2.1 Models

The standard IVIM model equation is:

$$\bar{S}=fe^{-bd_{\mathrm{iso}}^{*}}+\left(1-f\right)e^{-bd_{\mathrm{iso}}}$$

where $\bar{S}$ is the dMRI signal averaged across gradient directions, f is the perfusion fraction, $d_{\mathrm{iso}}^{*}$ is the perfusion‐related (isotropic) pseudo‐diffusivity, and $d_{\mathrm{iso}}$ is the diffusion‐related isotropic diffusivity. In this paper, we refer to this model as “standard IVIM” but note that it could also be termed “ball‐ball” as it has two isotropic compartments, or “balls” in microstructure modeling terminology.

Here we introduce two anisotropic IVIM spherical mean (SM) approaches, based on the stick‐zeppelin and zeppelin‐zeppelin models that were previously proposed for placental dMRI [13]. We selected these models as their standard (i.e., directional) versions were shown to best explain the data in this earlier study [13]. These are two‐compartment models consisting of “sticks”—that is, tensors with diffusion constrained to only one direction [17]—and “zeppelins”—that is, cylindrically constrained tensor [18]—components.

The spherically averaged signal for a stick compartment is [19]:

$$\bar{S_{s}}\left(d_{\mathrm{par}}\right)=\frac{\sqrt{\pi }\;\mathrm{erf}\left(\sqrt{bd_{\mathrm{par}}}\right)}{2\sqrt{bd_{\mathrm{par}}}}$$

where erf() is the error function, *d*_par_ is the parallel diffusivity, $\bar{S_{s}}$ is the spherically averaged signal and *b* is the *b* value. We use the spherically averaged stick compartment to model the perfusion‐related signal component; similar to its application in the VERDICT model [20].

The spherically averaged zeppelin signal is [16, 19]:

$$\bar{S_{z}}\left(d_{\text{perp}},d_{\mathrm{par}}\right)=\exp\left(-bd_{\text{perp}}\right)\frac{\sqrt{\pi }\;\mathrm{erf}\left(\sqrt{b\left(d_{\mathrm{par}}-d_{\text{perp}}\right)}\right)}{\sqrt{b\left(d_{\mathrm{par}}-d_{\text{perp}}\right)}}$$

where $d_{\text{perp}}$ is the perpendicular diffusivity. We use the spherically averaged zeppelin to model both the perfusion‐ and diffusion‐related signal components.

The signal for the spherical mean zeppelin‐zeppelin model, which we denote SM zeppelin‐zeppelin from hereon, is:

$$\bar{S}=f\bar{S_{z}}\left(d_{\text{perp}}^{*},d_{\mathrm{par}}^{*}\right)+\left(1-f\right)\bar{S_{z}}\left(d_{\text{perp}},d_{\mathrm{par}}\right)$$

and the signal for the spherical mean stick‐zeppelin model, which we denote SM stick‐zeppelin, is:

$$\bar{S}=f\bar{S_{s}}\left(d_{\mathrm{par}}^{*}\right)+\left(1-f\right)\;\bar{S_{z}}\left(d_{\text{perp}},d_{\mathrm{par}}\right)$$

Figure 1 schematically represents each spherical mean model and compares them to the standard IVIM and diffusion tensor models.

**FIGURE 1:**
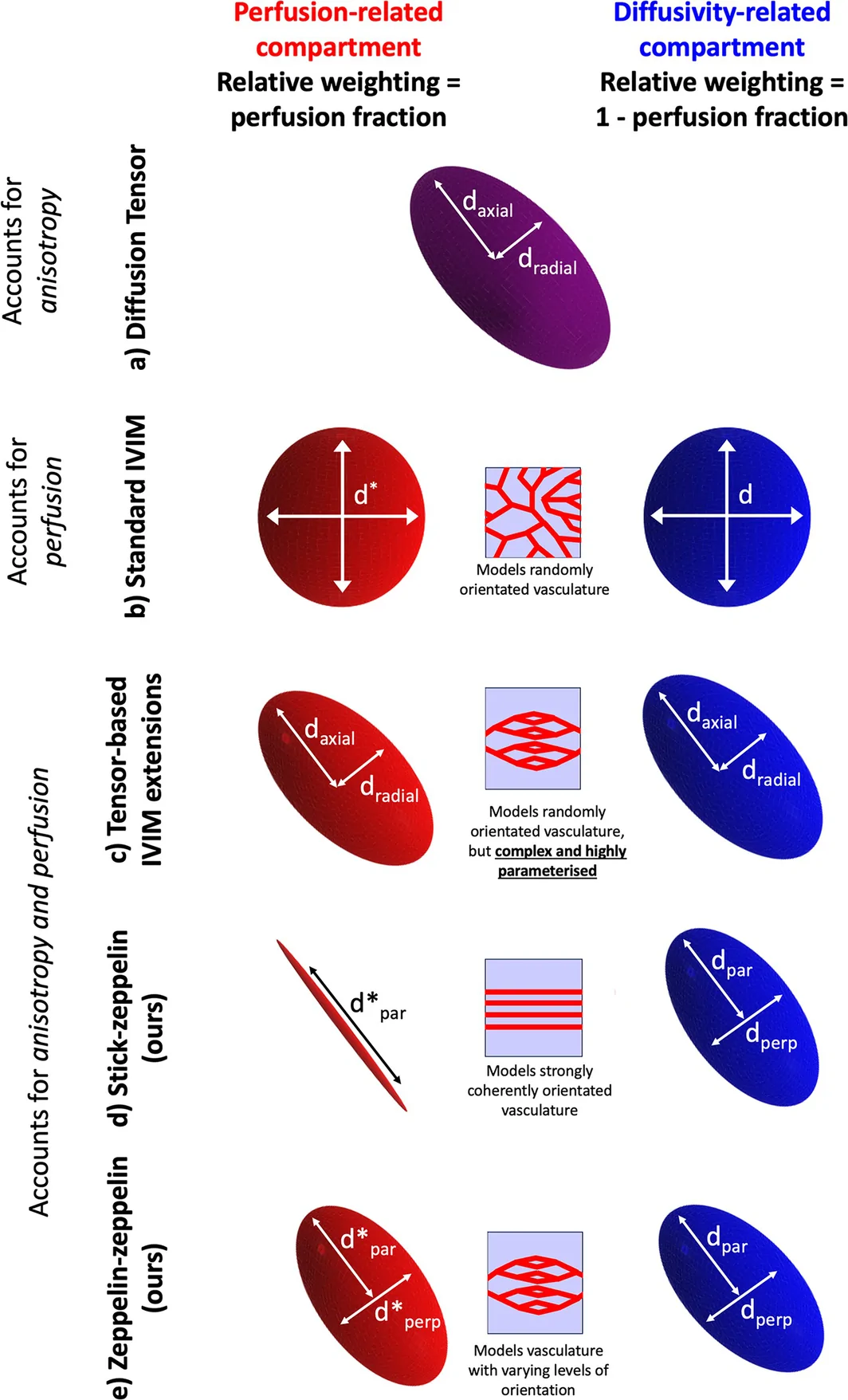
Schematic of the models employed, alongside diffusion tensor model for comparison. Red denotes a compartment designed to capture perfusion‐related signal components; blue denotes a compartment designed to capture diffusion‐related signal components. (a) Diffusion tensor, which is colored purple as it does not separate perfusion‐ and diffusion‐related components; (b) Standard IVIM model [1]; (c) tensor‐related IVIM extensions such as [4, 7, 8, 9]; (d–e) stick‐zeppelin and zeppelin‐zeppelin models as in [13]. We use spherical mean versions of the stick‐zeppelin and zeppelin‐zeppelin models in this paper.

### 2.2 Data

Pregnant participants gave informed consent to undergo dMRI of the placenta as part of an ethically approved study (DAPHNE, REC 22/YH/0144). Here, we include data from 14 participants with normal pregnancy outcomes, defined as a pregnancy ending in the birth of a normal birthweight baby between 37 and 41 weeks' gestation with no medical complications during pregnancy. Six participants had a single scan, four participants had two scans, and four participants had three scans, giving a total of 26 scans. All scans were performed between 22 and 38 weeks of pregnancy.

Participants were scanned on a Siemens Vida scanner at 3T using the Body 18 coil, utilizing a previously published dMRI protocol that was optimized for the placenta [21]. Briefly, the protocol has *b* = 5, 10, 25, 50, 100, 200, 400, 600, 1200, 1600 s/mm^2^ shells with three gradient directions, in addition to *b* = 18, 36, 800 s/mm^2^ shells with 8, 7, and 15 directions, respectively. The gradient directions are jointly optimized across all shells to maximize angular coverage as previously described [22]. There are six *b* = 0 volumes and 66 volumes in total. Other imaging parameters were TE = 83 ms, TR = 3300 ms, Grappa acceleration factor 2, partial Fourier 6/8, voxel size = 3 × 3 × 15 mm, image matrix 128 × 128 × 9. The total acquisition time was 3 min 7 s.

Data were pre‐processed with MP‐PCA denoizing [23], Rician bias correction [24], and Gibbs ringing removal [25]. We manually defined two regions of interest (ROIs): one containing the placenta and one containing the adjacent uterine wall.

### 2.3 Model Fitting

We fit the standard IVIM (i.e., ball‐ball) model voxelwise using Dmipy's [26] two‐stage brute2fine method, fitting all *b* values simultaneously (i.e., not segmented IVIM fitting).

We fit the SM stick‐zeppelin and SM zeppelin‐zeppelin models voxelwise to the direction averaged dMRI data using modified dmipy [26] code (available at github.com/PaddySlator/dmipy). We used dmipy's brute2fine option, a two‐stage fit comprising a grid search then a nonlinear optimization for all fits. We also fit the directional versions of the stick‐zeppelin and zeppelin‐zeppelin models [13] in the same way. For SM stick‐zeppelin we calculated the FA of the diffusion‐related zeppelin compartment, and for SM zeppelin‐zeppelin we calculated the FA of the perfusion‐ and diffusion‐related zeppelin compartments.

All models were fit assuming Gaussian noise since we had corrected the Rician offset. We constrained the parameters to the following ranges: perfusion fractions (0–1), $d_{\mathrm{iso}}$ (0.1–30 μm^2^ ms^−1^), $d_{\mathrm{par}}$ (0.1–30 μm^2^ ms^−1^), $d_{\text{perp}}$ (0.1–30 μm^2^ ms^−1^), $d_{\mathrm{iso}}^{*}$ (30–300 μm^2^ ms^−1^), $d_{\mathrm{par}}^{*}$ (30–300 μm^2^ ms^−1^), $d_{\text{perp}}^{*}$ (30–300 μm^2^ ms^−1^). We also impose that $d_{\mathrm{par}}>d_{\text{perp}}$ and $d_{\mathrm{par}}^{*}>d_{\text{perp}}^{*}$. We chose a diffusion‐perfusion cut off of 30 μm^2^ ms^−1^, which is higher than the 3 μm^2^ ms^−1^ threshold (approximately the diffusivity of free water) typically used in standard IVIM. Our objective was to separate “true” perfusion—that is, blood flowing in capillaries and larger vessels—from all other signal components. In the placenta, the latter likely includes slowly flowing maternal blood percolating through the intervillous space, which may exhibit apparent diffusivities above 3 μm^2^ ms^−1^, as suggested by previous work [13, 27]. Based on these findings, we considered 30 μm^2^ ms^−1^ to be a reasonable threshold for separating these components. However, we acknowledge that this choice affects the interpretation of these components, moving away from the traditional IVIM separation of “perfusion” and “tissue.” For example, the diffusivity related to this compartment might be more accurately described as “diffusion‐ and percolating maternal blood‐related diffusivity.” For simplicity, however, we refer to this throughout as “diffusion‐related diffusivity” but encourage readers to keep this distinction in mind when interpreting the results.

We calculated the Bayesian information criterion (BIC) for the SM stick‐zeppelin, SM zeppelin‐zeppelin, and standard IVIM models in each voxel using the simplified BIC equation that assumes Gaussian noise:

$${\mathrm{BIC}}_{\text{model}}=n\;\log\left({\mathrm{MSE}}_{\text{model}}\right)+k\log\left(n\right)$$

where ${\mathrm{MSE}}_{\text{model}}$ is the mean squared error, n is the number of independent measurements (i.e., the number of datapoints in the direction‐averaged data, which is 14 in our case), and k is the number of independent model parameters, that is, 3 for standard IVIM, 4 for SM stick‐zeppelin, 5 for SM zeppelin‐zeppelin. We do not include the directional stick‐zeppelin and directional zeppelin‐zeppelin models in the BIC calculation, as the number of independent datapoints it fits to is higher (since it uses all directions), so a BIC comparison would not be meaningful.

## 3 Results

We first display the array of maps for all models that are outputted for each scan (Figure 2). We then show a range of parameter maps grouped by type for a representative sample of participants over gestational age in Figures 3, 4, 5, 6, 7. Figure 8 displays the relationships between model parameters and gestational age. Figure 9 displays which model has the lowest BIC, and hence explains the data best in each voxel.

**FIGURE 2:**
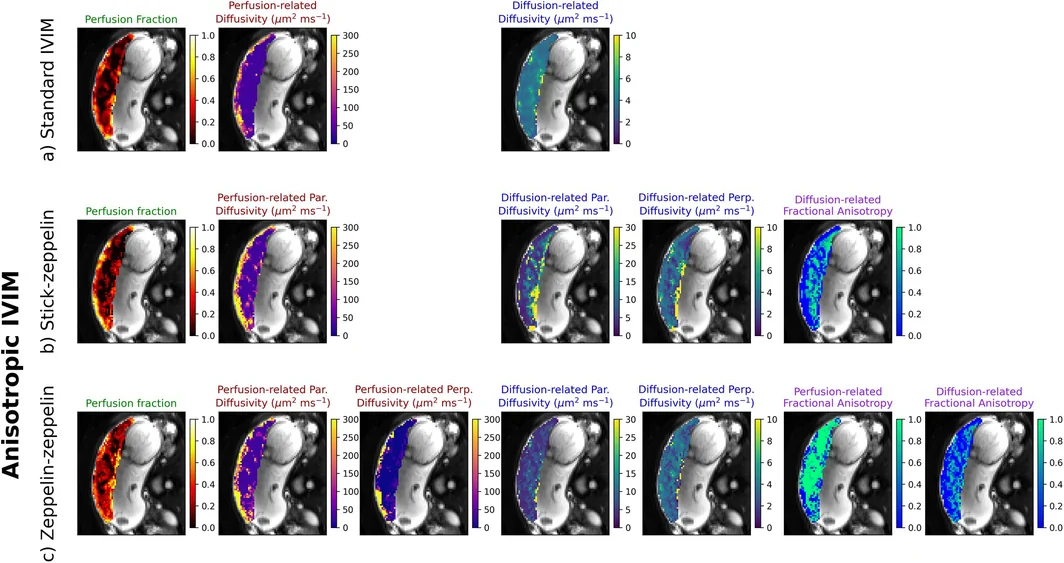
Parameter maps for a single participant with gestational age 28.28 weeks for (a) standard IVIM model; (b) SM stick‐zeppelin model; (c) SM zeppelin‐zeppelin model. Subplot titles are color‐coded by parameter type, green = perfusion fractions, dark red = perfusion‐related diffusivities, blue = diffusion‐related diffusivities, and purple = fractional anisotropies.

**FIGURE 3:**
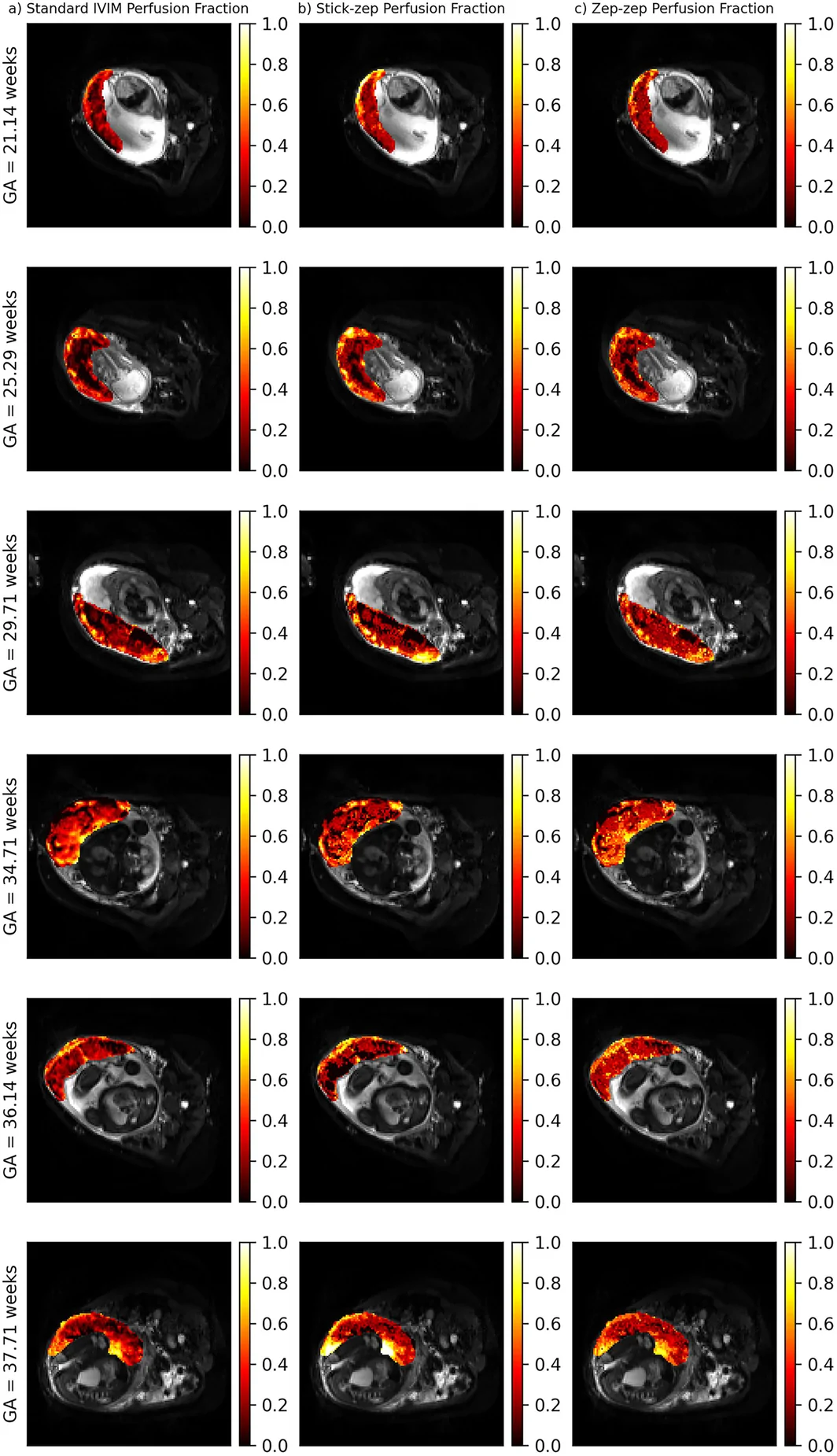
Perfusion fraction maps for (a) standard IVIM (i.e., ball‐ball), (b) SM stick‐zeppelin, and (c) SM zeppelin‐zeppelin models for six participants selected to cover a range of gestational ages (GA).

**FIGURE 4:**
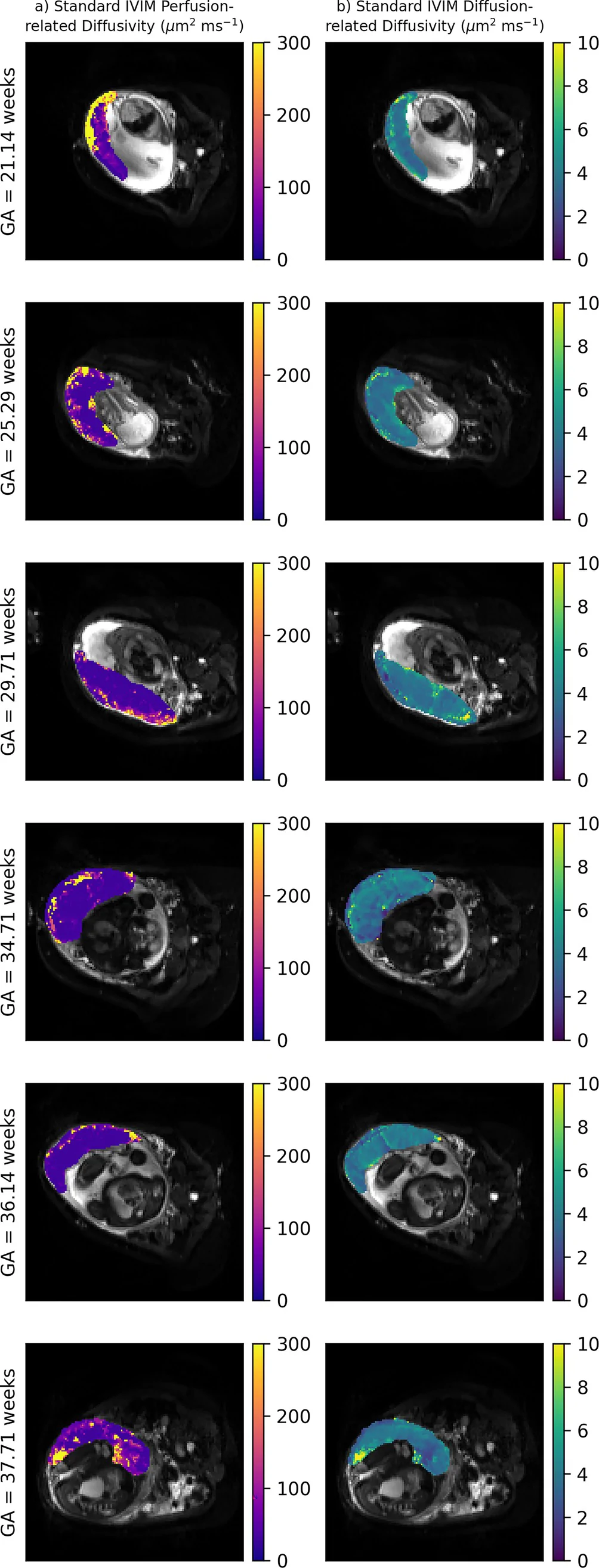
Isotropic diffusivity maps for (a) ball‐ball (i.e., standard IVIM) and (b) tensor models for six participants selected to cover a range of gestational ages (GA).

**FIGURE 5:**
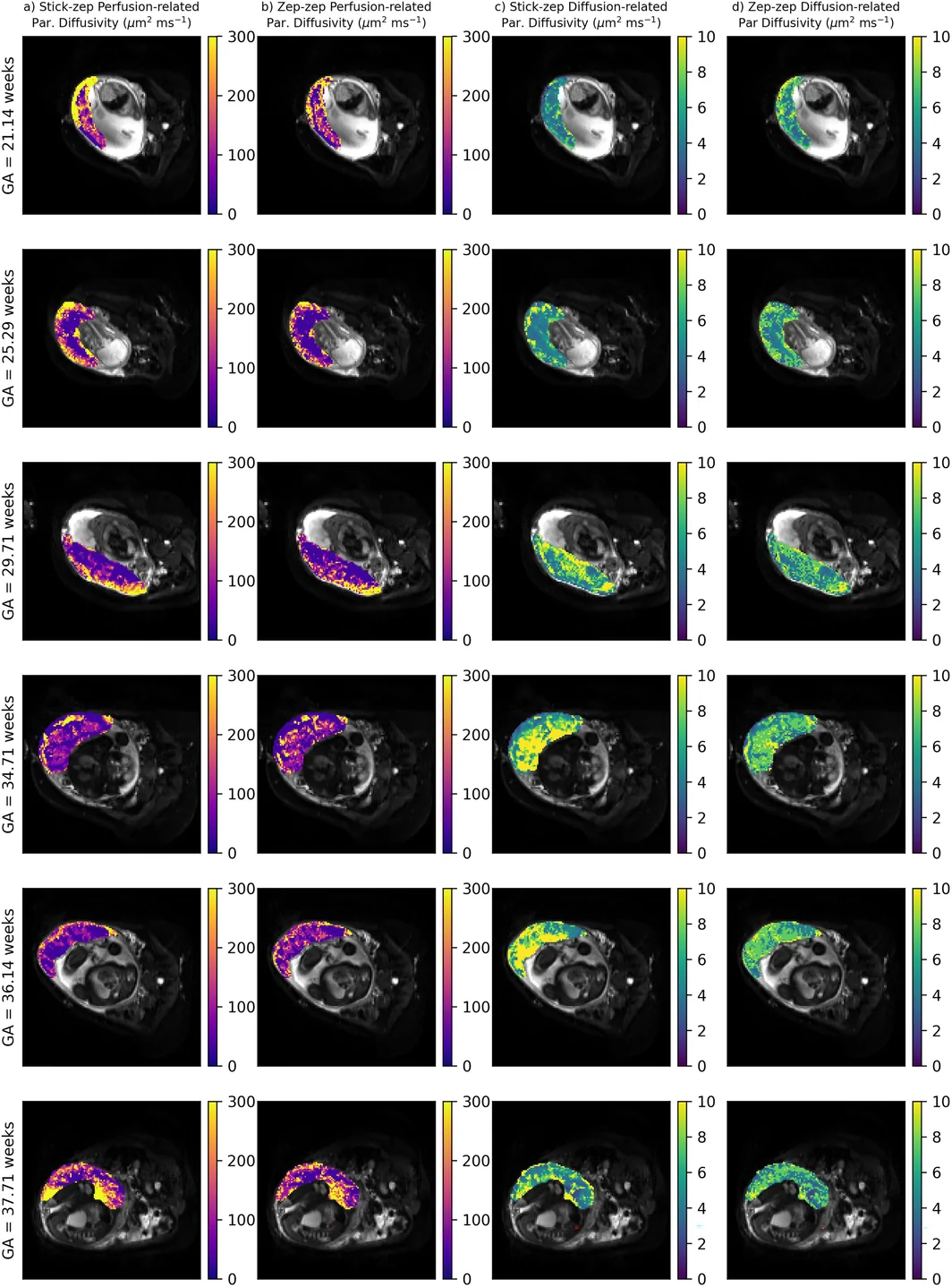
Perfusion‐ and diffusion‐related parallel diffusivity maps for SM stick‐zeppelin and SM zeppelin‐zeppelin models for six participants selected to cover a range of gestational ages (GA).

**FIGURE 6:**
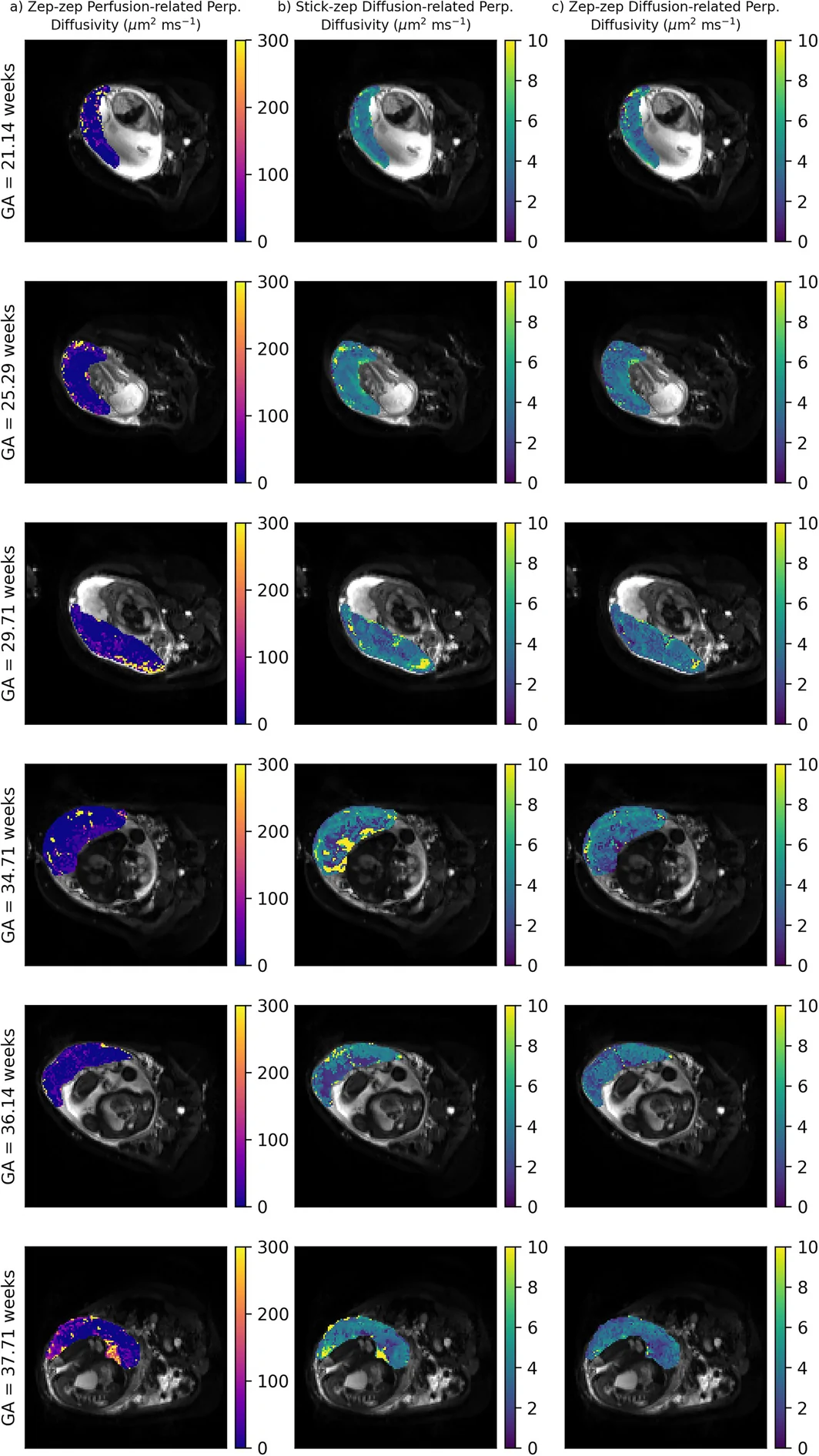
Perpendicular diffusivity maps for SM stick‐zeppelin and SM zeppelin‐zeppelin models for six participants selected to cover a range of gestational ages (GA).

**FIGURE 7:**
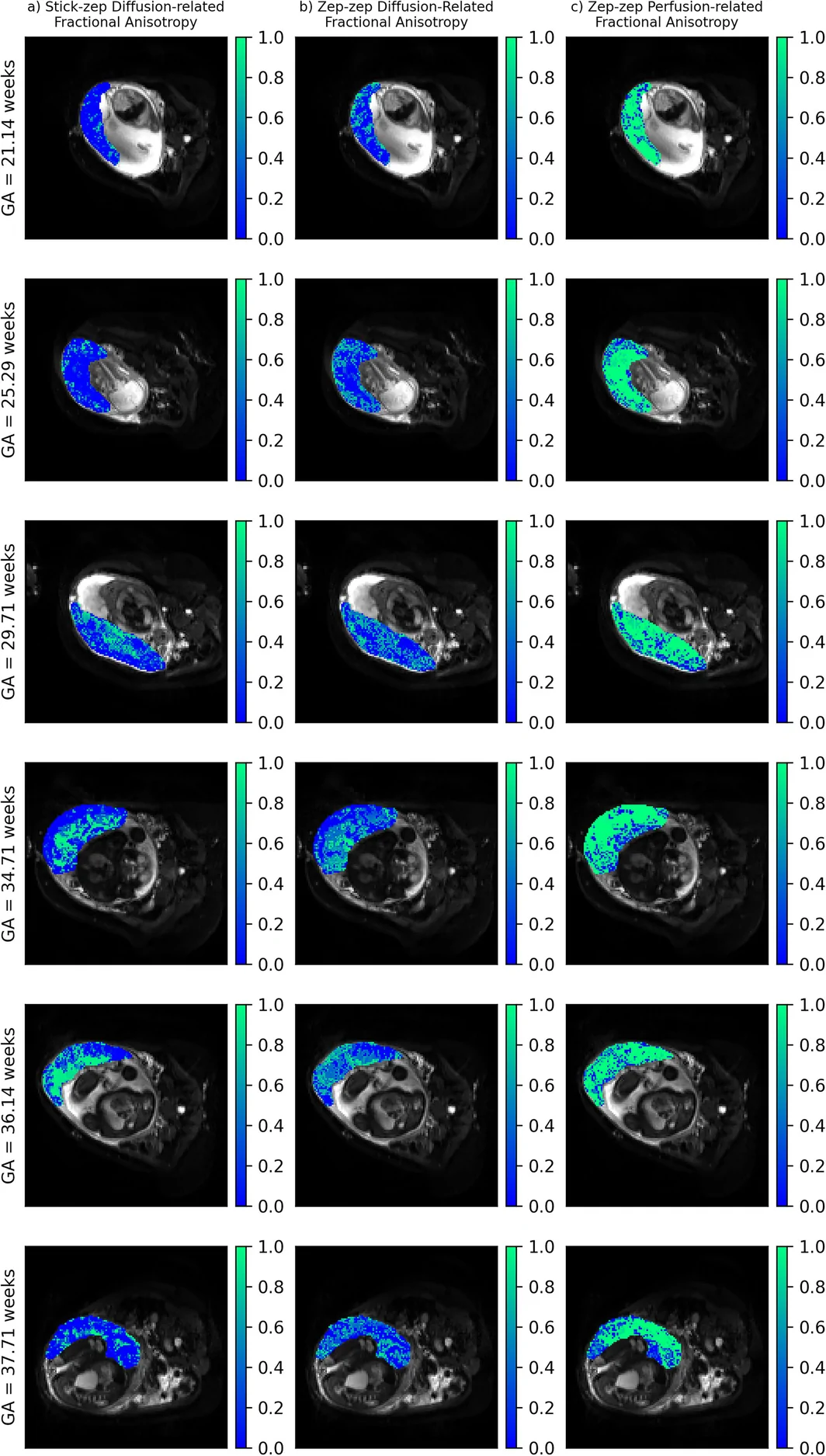
Fractional anisotropy maps for SM stick‐zeppelin and SM zeppelin‐zeppelin models for six participants selected to cover a range of gestational ages (GA).

**FIGURE 8:**
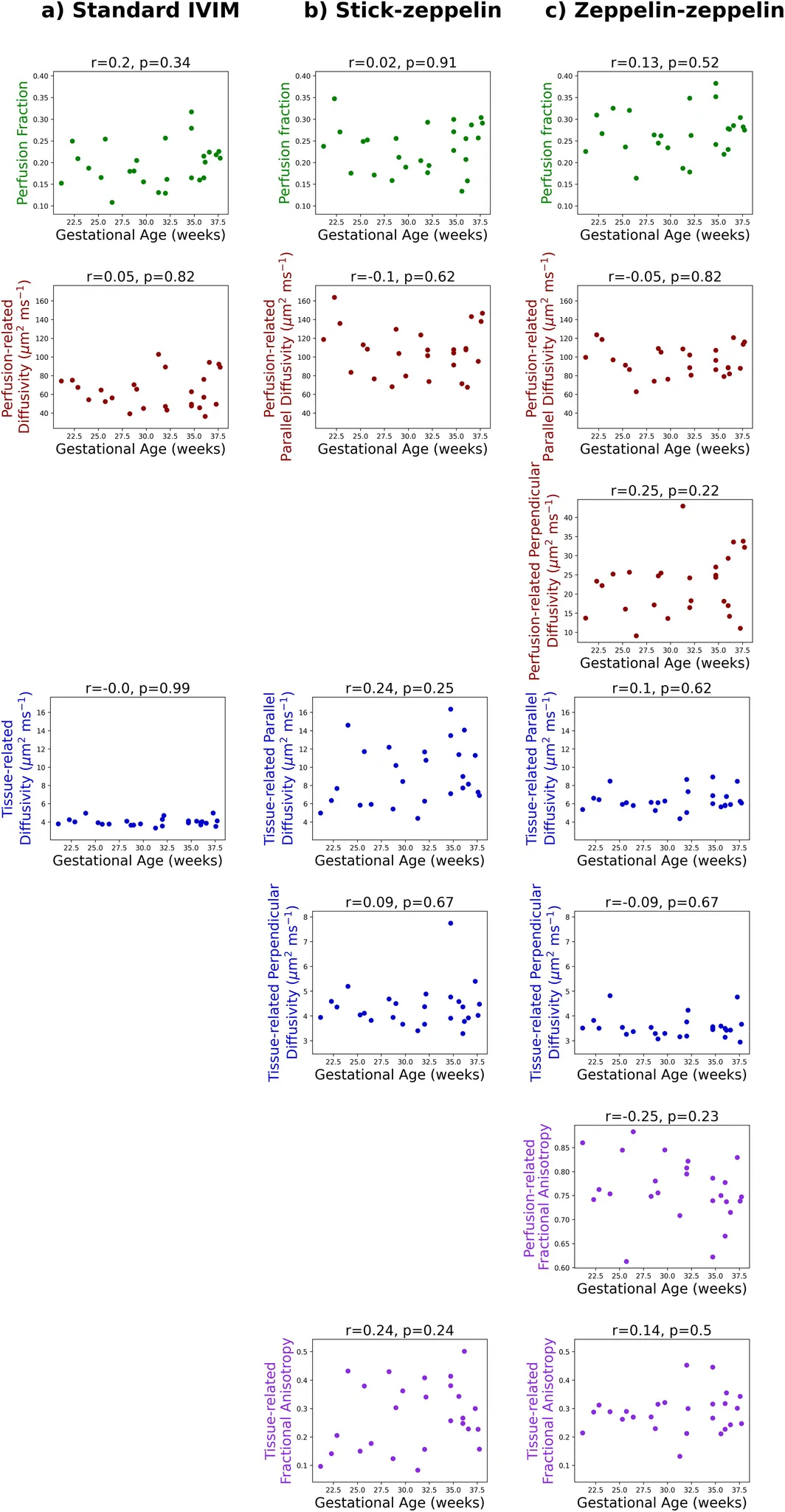
Mean parameter values within placenta ROIs plotted against gestational age for (a) standard IVIM; (b) SM stick‐zeppelin; and (c) SM zeppelin‐zeppelin. *r* denotes Pearson's correlation coefficient. See Figure S6 for uterine wall ROI version.

**FIGURE 9:**
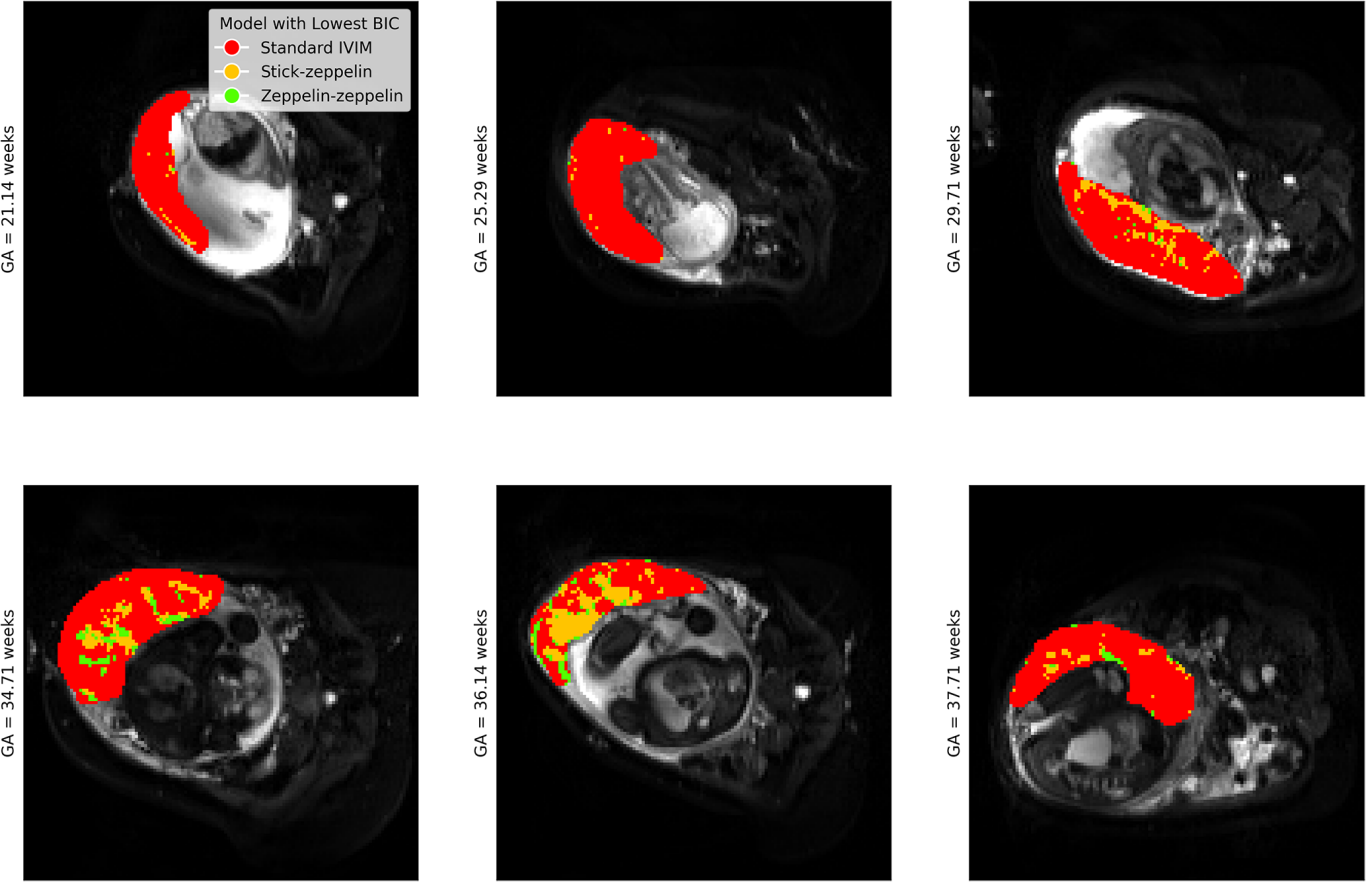
Maps showing which model has the lowest Bayesian information criterion (BIC), and hence is best supported by the data, in each voxel.

Figure 2 presents maps from a single scan at 28 weeks' gestation. The first row shows standard IVIM maps, while the bottom two rows display the new maps generated by the spherical mean anisotropic IVIM models. Both SM stick‐zeppelin and SM zeppelin‐zeppelin separate the diffusion‐related diffusivity IVIM map into parallel and perpendicular diffusivity maps, and the corresponding diffusion‐related FA map. The SM zeppelin‐zeppelin model additionally separates the perfusion‐related pseudo‐diffusivity into parallel and perpendicular pseudo‐diffusivity maps and the corresponding perfusion‐related fractional anisotropy map. Notably, there are clear differences in the perfusion‐ and diffusion‐related FA maps. Additionally, considering the anisotropy in the signal results in clear differences in the perfusion fraction maps for SM stick‐zeppelin and SM zeppelin‐zeppelin compared to standard IVIM.

Figure 3 shows the perfusion fraction maps for IVIM, SM stick‐zeppelin, and SM zeppelin‐zeppelin models; these maps inform on the relative volume fraction of each voxel that is accounted for by perfusing blood. For all models, the maps show high perfusion fraction at the boundaries of the placenta—both around the boundary with the uterine wall and the chorionic plate. This is consistent with the literature [13] and suggests that these areas are highly vascularized. There are some differences in the maps between the three different models, likely due to the SM models additionally accounting for anisotropy.

Figure 4 shows isotropic diffusivity maps, which quantify diffusivity without considering directionality, from the standard IVIM model. Figure 5 shows parallel diffusivity maps, which quantify diffusivity parallel to the primary direction of a tensor‐like structure. These maps are derived from the SM stick‐zeppelin and SM zeppelin‐zeppelin models. Figure 6 shows perpendicular diffusivity maps, which quantify diffusivity perpendicular to the primary direction of a tensor‐like structure. These maps are derived from the SM stick‐zeppelin and SM zeppelin‐zeppelin models. Figure 7 shows fractional anisotropy maps from the SM stick‐zeppelin and SM zeppelin‐zeppelin models.

Figure 8 plots the ROI‐averaged parameter values against gestational age for each participant, showing the extent to which these parameters capture diffusion‐ and perfusion‐related changes over gestation. Most parameters have low correlation coefficients, and none have statistically significant trends over gestation. In the uterine wall ROI (Figure S6), the parameter most strongly correlated with gestation is the standard IVIM diffusion‐related diffusivity (*r* = −0.27). In the placenta ROI, the highest correlations are observed for the standard IVIM perfusion fraction (*r* = 0.20), SM zeppelin–zeppelin perfusion‐related perpendicular pseudo‐diffusivity (*r* = 0.25) and perfusion‐related fractional anisotropy (*r* = −0.25), as well as SM stick–zeppelin diffusion‐related parallel diffusivity (*r* = 0.24) and diffusion‐related fractional anisotropy (*r* = 0.24).

Figure 9 assesses which IVIM model best explains the data across the whole maps. It does this by highlighting which model has the lowest value of the BIC in each voxel. The standard, isotropic IVIM model explained the data best in most voxels, although spherical mean anisotropic IVIM models explained the data better in some voxels, particularly within central placental regions.

## 4 Discussion

We introduce two spherical mean anisotropic IVIM models, SM stick‐zeppelin and SM zeppelin‐zeppelin. We apply these models to placental dMRI and compare them with standard IVIM models. Our experiments yielded the following encouraging observations.

1. Spherical mean anisotropic IVIM models generate parameter maps that disentangle perfusion‐ and diffusion‐related diffusivity and anisotropy.
2. Spherical mean anisotropic IVIM parameter maps display similar but subtly different patterns to standard IVIM.
3. Spherical mean anisotropic IVIM parameter maps explain the data better than standard IVIM in a considerable proportion of the placenta.

These observations suggest that these two models have potential for separately mapping the diffusivity and anisotropy of tissue microstructure and vascular microcirculation. A key advantage of the spherical mean approach over existing anisotropic IVIM methods is the simplicity and efficiency of model fitting, particularly in comparison to directional models.

Compared to standard IVIM, the additional maps derived from SM stick‐zeppelin and SM zeppelin‐zeppelin include parallel diffusivities for both perfusion‐ and diffusion‐related compartments, perpendicular diffusivities for these compartments (with perfusion‐related perpendicular diffusivities provided only by SM zeppelin‐zeppelin), and separate FA maps for perfusion‐ and diffusion‐related compartments (with perfusion‐related FA maps provided only by SM zeppelin‐zeppelin). These maps share the key characteristic of being independently sensitive to anisotropy in both perfusion‐ and diffusion‐related signal components. While some parameter maps from SM stick‐zeppelin and SM zeppelin‐zeppelin share names with maps from existing models, such as the perfusion fraction of standard IVIM, they are distinct because these models additionally account for anisotropy in the signal.

SM zeppelin‐zeppelin and SM stick‐zeppelin perfusion fraction maps show comparable patterns to standard IVIM perfusion fraction maps (Figure 3). The SM stick‐zeppelin model potentially overestimates the perfusion fraction compared to SM zeppelin‐zeppelin and standard IVIM, particularly in the uterine wall, which is a highly anisotropic structure (see Figure 7, first row). This discrepancy may be because SM stick‐zeppelin assumes maximally anisotropic perfusion, so highly anisotropic diffusion‐related features could be pushed into the perfusion compartment. This suggests that SM zeppelin‐zeppelin may be a preferable model due to its ability to model varying levels of anisotropy and the lack thereof.

The anisotropy‐related SM stick‐zeppelin and SM zeppelin‐zeppelin maps offer complementary insights to maps obtained from standard IVIM. For instance, the parallel diffusivities (both perfusion‐ and diffusion‐related) from the SM stick‐zeppelin and SM zeppelin‐zeppelin models (Figure 5) display greater contrast in central placenta regions compared to standard IVIM diffusivity and pseudo‐diffusivity maps (Figure 4). Additionally, Figure 7 shows that SM stick‐zeppelin FA maps highlight interesting patterns on the chorionic half of the placenta, with the stick compartment in SM stick‐zeppelin models potentially enabling subtle FA features in diffusion‐related compartments to be revealed. Zeppelin‐zeppelin perfusion FA maps are uniformly high, potentially reflecting the strong anisotropy of placental vasculature. However, the elevated FA values may also be influenced by noise in signals from low *b* values that are very close to each other. We also note that values remain uniformly high in areas where the zeppelin‐zeppelin perfusion fraction is very low or zero. In these areas, the perfusion‐related FA has limited physiological meaning, as it is derived from only a small fraction of the total signal. Future studies using protocols with a greater number of low *b* values may help determine whether the elevated perfusion‐related FA observed in these regions reflects a genuine physiological phenomenon or is primarily driven by noise.

To assess whether they capture similar information, we compare spherical mean and directional implementations of the stick‐zeppelin and zeppelin‐zeppelin models in Figures S1–S5. The corresponding parameter maps exhibit similar spatial patterns, suggesting that both formulations capture broadly the same information. However, there are some slight differences between the maps. For example, interestingly, the SM models are more likely to find zero or very low perfusion fraction than the directional models; this may reflect that some directionally coherent flow is not picked up by the SM models and is put into the diffusion‐related compartment instead.

The mean dMRI parameter values do not exhibit strong trends against gestational age (Figure 8), which contrasts with existing literature. Previous studies have reported variable patterns in apparent diffusion coefficient (ADC) and perfusion fraction across gestation, with some showing increases in ADC [28] and perfusion fraction [29, 30, 31, 32], while others report decreases in ADC [29, 31] and perfusion fraction [33, 34, 35]. Several factors could account for the weaker trends observed in our parameters compared to those in prior studies. Most importantly, as detailed in Methods, we use a diffusion‐perfusion cut off value of 30 μm^2^ ms^−1^ rather than the conventional IVIM threshold of 3 μm^2^ ms^−1^. This choice will inevitably influence the overall trends observed in the model parameters. Moreover, this study only includes 26 scans from 14 participants and may be underpowered to detect gestational changes—more data is required to draw firm conclusions.

However, if there is indeed no underlying trend, one possible explanation is the difference in acquisition sequences; for instance, our use of anisotropic voxels may affect the estimation of various diffusion parameters. Another factor may be our use of whole organ ROIs. Our maps show that both the placenta and uterine wall have heterogeneous spatial patterns, so averaging across whole organ ROIs may obscure changes occurring within smaller subregions. Alternatively, the lack of strong trends may suggest that our approach can factor out microcirculatory and microstructural changes across gestation. This could potentially enable clearer comparisons between cases and controls, as strong gestational‐age dependence in a parameter may confound group differences. Although we could not investigate this in the current study, as we only include control participants, future work will address this question. Regarding potential clinical impact, we speculate that our approach may be most relevant in conditions where the standard IVIM perfusion fraction has shown promise as a biomarker, including fetal growth restriction [36], preeclampsia [36], and vascular malperfusion [37]. Unlike standard IVIM's isotropic perfusion‐ and diffusion‐related signal components, our approach accounts for their anisotropy and may therefore provide a more accurate perfusion fraction estimate.

Figure 10 shows that spherical mean anisotropic IVIM models explain the data better than the standard IVIM model in up to 30% and 15% of the voxels in the placenta and uterine wall masks respectively, according to the BIC. This proportion is highest in the placenta in late gestation, with 20%–30% of placenta voxels better explained by anisotropic IVIM models. These findings suggest the presence of anisotropic vascular orientation and tissue microstructure in a substantial portion of the placenta, especially in later gestation. Furthermore, Figure 9 appears to show that voxels on the fetal side of the placenta are more likely to be described better by a SM model. This could reflect the fact that the fetal side of the placenta contains more microscopic anisotropy in perfusion structures such as tightly packed capillaries, which are well described by the SM models, whereas the maternal side contains more maternal blood flowing in intervillous space, which is sufficiently described by conventional IVIM. It is also possible that the performance of standard IVIM deteriorates at later gestational ages because it cannot capture the increasing microscopic anisotropy associated with the substantial rise in placental capillary density throughout gestation. As a result, apparent gestational trends in standard IVIM parameters may partly reflect the limitations of the model in representing the underlying microstructure, rather than genuine physiological changes in the tissue. Additionally, the fact that the standard IVIM model explains the data better in the uterine wall may indicate that anisotropic vascular orientation and tissue microstructure are more prominent in placental regions. This is consistent with emerging 3D histology studies showing evidence of flow anisotropy in the intervillous space [38].

**FIGURE 10:**
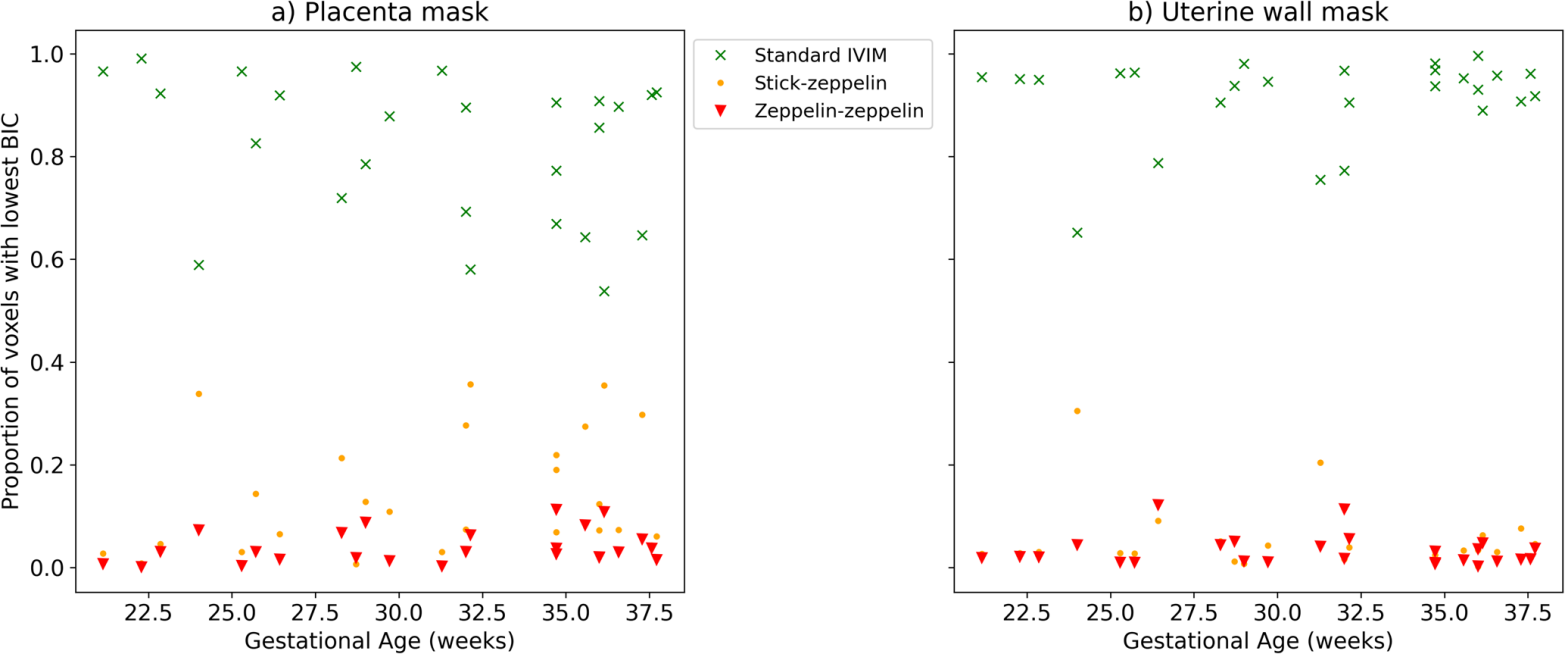
Plot showing proportion of voxels where each model has the lowest Bayesian information criterion (BIC) for (a) placenta and (b) uterine wall masks.

The uterine wall contains many coherently orientated fibers. Spherical mean models are designed to suppress the effects of such macroanisotropy, making them less sensitive to directional features like a single dominant fiber orientation. As a result a directional model may better explain the signal in this region, as was previously observed for full stick‐zeppelin and zeppelin‐zeppelin models [13]. However, direct comparisons between spherical mean and directional models are challenging, particularly using metrics such as BIC, since the models are fit to different numbers of independent data points.

A major advantage of spherical mean models is that they are much easier and faster to fit than directional anisotropic IVIM models. While the simpler standard IVIM model may be theoretically preferable in most voxels, the SM stick‐zeppelin and SM zeppelin‐zeppelin models could capture more detailed information relevant for clinical decision making in a feasible timescale. In particular, SM zeppelin‐zeppelin, which can represent both isotropic and anisotropic features in the perfusion and diffusion‐related signal components, offers added flexibility and robustness across different tissue structures.

Overall, our findings indicate that spherical mean anisotropic IVIM models are promising alternative options for analyzing dMRI data in structures with coherent vasculature, such as the placenta. We calculated the orientationally averaged dMRI signal using simple arithmetic averaging across gradient directions. However, this approach is not necessarily optimal [39]. In future, we will explore alternative methods for powder averaging, such as spherical harmonic‐based approaches. We did not perform motion correction, for example, for maternal breathing motion, as there are currently no off‐the‐shelf solutions for motion correction in diffusion MRI acquired during pregnancy. Therefore, voxel misregistration between dMRI volumes may have introduced fitting errors and confounded our results. Future work will address this limitation by developing a dedicated motion‐correction pipeline tailored to our acquisition protocol. Future work should also focus on testing the spherical mean anisotropic IVIM models in a larger study, including complicated pregnancies, to determine if the models can support clinical decisions during pregnancy. Future studies will also include test–retest acquisitions to assess intra‐subject repeatability, as previously demonstrated for anisotropic IVIM in the kidney [40], of both the protocol and the resulting quantitative maps. With a broader perspective, we aim to apply these models in other areas where anisotropic IVIM has shown promise, such as skeletal muscle, kidney, and heart, with the aim of discovering new biomarkers that can advance along the translational pipeline.

## 5 Conclusion

We introduce two spherical mean anisotropic IVIM models, SM stick‐zeppelin and SM zeppelin‐zeppelin, and apply them to placental dMRI. Spherical mean anisotropic IVIM models explain the data better than standard IVIM in a considerable proportion of voxels, suggesting that there are anisotropic vascular structures in a considerable proportion of the placenta that can be explored in future 3D histology studies. The parameters of these models include perfusion‐ and diffusion‐specific FA and diffusivity metrics and can be potential biomarkers for various diseases.

## Funding

This work was funded by the Wellcome Leap In Utero Program through the project “Multi‐model studies to understand pregnancy and prevent stillbirth”. The work was also supported by the Academy of Medical Sciences (Clinical Lecturer's starter grant for KD), Wellbeing of Women (post‐doctoral fellowship for KD), and the Engineering and Physical Sciences Research Council (EPSRC) (EP/V034537/1).

## Conflicts of Interest

The authors declare no conflicts of interest.

## Supporting information


**Figure S1:** Perfusion fraction maps for directional and spherical mean versions of stick‐zeppelin and zeppelin‐zeppelin models for six participants selected to cover a range of gestational ages.
**Figure S2:** Perpendicular diffusivity maps for directional and spherical mean versions of the stick‐zeppelin model for six participants selected to cover a range of gestational ages.
**Figure S3:** Perpendicular diffusivity maps for directional and spherical mean versions of the zeppelin‐zeppelin model for six participants selected to cover a range of gestational ages.
**Figure S4:** Parallel diffusivity maps for directional and spherical mean versions of the stick‐zeppelin model for six participants selected to cover a range of gestational ages.
**Figure S5:** Parallel diffusivity maps for directional and spherical mean versions of the zeppelin‐zeppelin model for six participants selected to cover a range of gestational ages.
**Figure S6:** As Figure 8 but for uterine wall ROI.

## Data Availability

The data that support the findings of this study are available from the authors upon reasonable request and subject to signing of a data sharing agreement. The dataset is owned by the University of Manchester and was collected as part of the Wellcome Leap In Utero Program project “Multi‐model studies to understand pregnancy and prevent stillbirth”. Researchers interested in accessing the data should contact the corresponding author (PJS) and project PI (AEPH) in the first instance.
