# Supplementary material for: Simplified Anisotropic IVIM Using Spherical Means and an Application in the Placenta
Source: Magn Reson Med. 2026 Sep 20;97(1):378–93. doi: 10.1002/mrm.70598 (PMC13746067; doi:10.1002/mrm.70598)
Supplement: Supplementary file 1 [file MRM-97-378-s001.docx]

Supporting Information

Supporting Figure 1: Perfusion fraction maps for directional and spherical mean versions of stick-zeppelin and zeppelin-zeppelin models for six participants selected to cover a range of gestational ages.

Supporting Figure 2: Perpendicular diffusivity maps for directional and spherical mean versions of the stick-zeppelin model for six participants selected to cover a range of gestational ages.

Supporting Figure 3: Perpendicular diffusivity maps for directional and spherical mean versions of the zeppelin-zeppelin model for six participants selected to cover a range of gestational ages.

Supporting Figure 4: Parallel diffusivity maps for directional and spherical mean versions of the stick-zeppelin model for six participants selected to cover a range of gestational ages.

Supporting Figure 5: Parallel diffusivity maps for directional and spherical mean versions of the zeppelin-zeppelin model for six participants selected to cover a range of gestational ages.

Supporting Figure 6: As Figure 8 but for uterine wall ROI.
